# *Akkermansia muciniphila*-Derived Extracellular Vesicles as a Mucosal Delivery Vector for Amelioration of Obesity in Mice

**DOI:** 10.3389/fmicb.2019.02155

**Published:** 2019-10-01

**Authors:** Fatemeh Ashrafian, Arefeh Shahriary, Ava Behrouzi, Hamid Reza Moradi, Shahrbanoo Keshavarz Azizi Raftar, Arezou Lari, Shima Hadifar, Rezvan Yaghoubfar, Sara Ahmadi Badi, Shohre Khatami, Farzam Vaziri, Seyed Davar Siadat

**Affiliations:** ^1^Department of Mycobacteriology and Pulmonary Research, Pasteur Institute of Iran, Tehran, Iran; ^2^Microbiology Research Center, Pasteur Institute of Iran, Tehran, Iran; ^3^Department of Histology and Embryology Group, Basic Sciences, Faculty of Veterinary Medicine, Shiraz University, Shiraz, Iran; ^4^Systems Biomedicine Unit, Pasteur Institute of Iran, Tehran, Iran; ^5^Department of Biochemistry, Pasteur Institute of Iran, Tehran, Iran

**Keywords:** gut microbiota, *Akkermansia muciniphila*, extracellular vesicles, toll-like receptors, tight junction, peroxisome proliferator-activated receptors, Angplt4, obesity

## Abstract

Recent evidence suggests that probiotics can restore the mucosal barrier integrity, ameliorate inflammation, and promote homeostasis required for metabolism in obesity by affecting the gut microbiota composition. In this study, we investigated the effect of *Akkermansia muciniphila* and its extracellular vesicles (EVs) on obesity-related genes in microarray datasets and evaluated the cell line and C57BL/6 mice by conducting RT-PCR and ELISA assays. *A. muciniphila*-derived EVs caused a more significant loss in body and fat weight of high-fat diet (HFD)-fed mice, compared with the bacterium itself. Moreover, treatment with *A. muciniphila* and EVs had significant effects on lipid metabolism and expression of inflammatory markers in adipose tissues. Both treatments improved the intestinal barrier integrity, inflammation, energy balance, and blood parameters (i.e., lipid profile and glucose level). Our findings showed that *A. muciniphila*-derived EVs contain various biomolecules, which can have a positive impact on obesity by affecting the involved genes. Also, our results showed that *A. muciniphila* and its EVs had a significant relationship with intestinal homeostasis, which highlights their positive role in obesity treatment. In conclusion, *A. muciniphila*-derived EVs can be used as new therapeutic strategies to ameliorate HFD-induced obesity by affecting various mechanisms.

## Introduction

Obesity, defined as the excessive accumulation of body fat, is considered a global public health problem in children and adolescents. According to reports by the World Health Organization in 2016, up to 650 million adults suffer from obesity worldwide. In the past, obesity was only a common problem in developed countries, while today, it also occurs in developing countries (WHO, 2016). Increased body mass index (BMI) is considered a major risk factor for diseases associated with obesity, such as metabolic syndrome, cardiovascular disease, type II diabetes, and cancer (Emerging Risk Factors Collaboration, 2011; Singh et al., 2013; Lauby-Secretan et al., 2016).

Obesity is generally a multifactorial phenomenon. The gut microbiota is one of the factors, which has been recently considered in obesity (Bouter et al., 2017). The gut microbiota is defined as trillions of microbes, which colonize in the gastrointestinal tract, and play a crucial role in human health (Zhang et al., 2015). Changes in the intestinal microbiota occur instantly after dietary changes, affecting numerous molecular pathways (David et al., 2014). Evidence shows that a high-fat diet (HFD) can cause an increase in the adipocyte size and improve weight gain. It can also result in the local inflammation of adipose tissues, which play an effective role in obesity through modulation of host metabolism by secretion of various hormones and cytokines (Turnbaugh et al., 2009; Grant and Dixit, 2015; Rastelli et al., 2018; Torres et al., 2019).

Obesity-induced adipose tissue dysfunction causes an increase in the level of free fatty acids and inflammatory cytokines and promotes hypertrophy and hyperplasia. In addition, HFD is associated with dysbiosis, mucosal barrier disruption, lipopolysaccharide (LPS) diffusion, and metabolic endotoxemia (Cani et al., 2007; Everard et al., 2013). On the other hand, accumulating evidence shows that probiotics can ameliorate obesity by improving the gut microbiota disruption, strengthening the gut barrier, alleviating inflammation, reducing body weight and fat storage, and inducing energy balance by affecting various mechanisms (Ejtahed et al., 2016, 2019; Plovier et al., 2017; Torres et al., 2019).

*Akkermansia muciniphila* is a beneficial gastrointestinal microbiota, which was recently introduced as a next-generation probiotic (Cani and de Vos, 2017). This mucin-degrading bacterium can influence the regulation of energy homeostasis and weight control (Everard et al., 2013; Plovier et al., 2017) and promote the intestinal barrier function (Everard et al., 2013; Reunanen et al., 2015; Ottman et al., 2017). Reduction in the abundance of this bacterium, as reported in multiple studies, indicates its pivotal role in the prevention of obesity (Everard et al., 2013; Schneeberger et al., 2015; Dao et al., 2016). It is known that the intestinal microbiota can modulate different signaling pathways by secreting extracellular vesicles (EVs). According to recent studies, not only EVs seem to be capable of passing the mucus and internalizing the epithelium, but they can also access the immune cells in the lamina propria as well as play a crucial role in the maintenance of immune and gut homeostasis by upregulation of tight junction proteins and modulation of immune responses (Lee et al., 2007; Fábrega et al., 2016; Ahmadi Badi et al., 2017; Behrouzi et al., 2018). Animal studies show that *A. muciniphila*-derived EVs were able to improve the intestinal barrier by increasing tight junctions (Chelakkot et al., 2018) and ameliorating inflammation caused by colitis (Kang C.-S. et al., 2013).

Involvement of the gut microbiota in weight changes is considered an important issue in obesity; therefore, it is essential to determine the association of microbiota with weight loss using probiotics. In the present study, nine HFD microarray datasets were selected. A meta-analysis of obesity-related genes was performed after batch effect removal. The anti-obesity and anti-inflammatory effects of *A. muciniphila* and its EVs were examined in epididymal adipose tissues (EAT). Also, colonic immunomodulatory properties of this bacterium and its EVs were assessed by measuring the concentration of cytokines in colon carcinoma cells (Caco-2) and evaluating the expression of cytokines and TLR-2/4 receptors in the colon of mice. Finally, the effects of *A. muciniphila* and its EVs on the intestinal barrier integrity, fat storage, and energy homeostasis were examined.

## Materials and Methods

### Preparation of *A. muciniphila*

*Akkermansia muciniphila MucT* (ATCC BAA-835) was obtained from the DSMZ institute (German collection of microorganisms and cell cultures). The bacterium was cultured in a basal mucin-based medium under the anaerobic conditions at 37°C for 3–7 days (Derrien et al., 2004). After growth, the bacterium was inoculated into brain heart infusion (BHI) Broth (Quelab, Canada) supplemented with 0.5% mucin (Sigma-Aldrich) with mild shaking (150 rpm) under the abovementioned conditions for 48 h until an OD_600_ of 1 was reached. Bacterial pellets were removed by centrifugation (11,000 *g* for 20 min) and washed twice with an anaerobic PBS. The remaining supernatant was used for EVs extraction. *A*. *muciniphila* suspension was immediately placed on ice and then used for cell culture treatment and oral administration in mice.

### EVs Isolation

After filtering the supernatant, EVs were extracted with ultracentrifuge at 200,000 *g* for 2 h at 4°C as previously described (Kang C.-S. et al., 2013). The pellets were resuspended in PBS and stored at –80°C. Scanning electron microscopy (SEM) was used to identify the morphology of its EVs and then the pattern of protein sample was assessed by SDS-PAGE. The presence of LPS in EVs was measured by LAL Chromogenic Endotoxin Quantitation Kit (Thermo Fisher Scientific, United States) according to the manufacturer’s instructions.

### Animal Experiments

**FIGURE 1:**
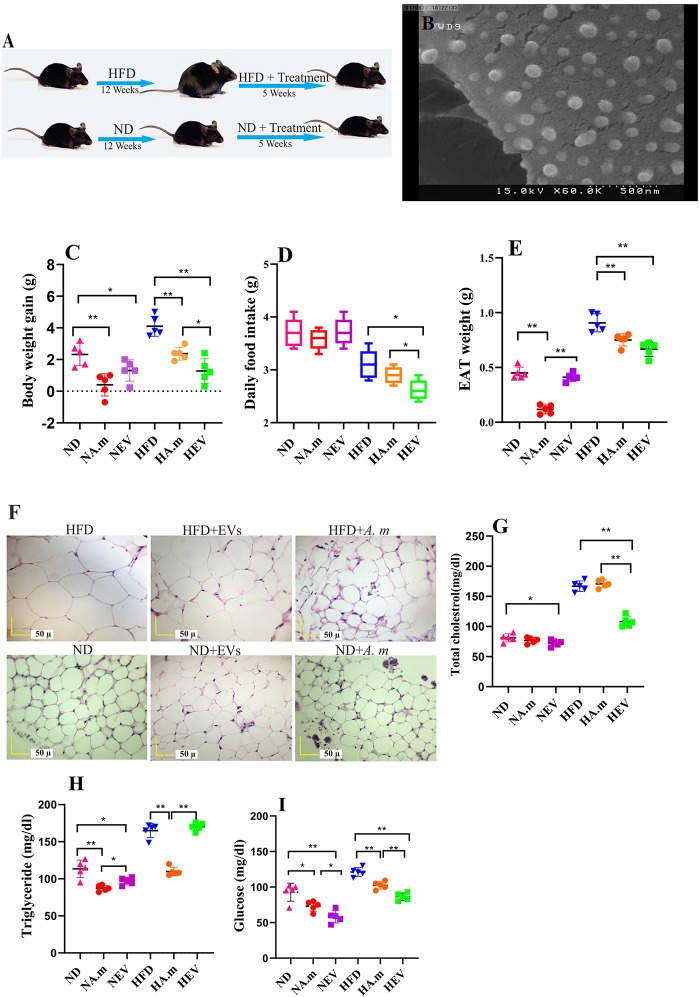
Morphologic characterization of EVs and impact of *Akkermansia muciniphila* and its EV administration on body and adipose weight, food intake, and blood parameters in both ND- and HFD-fed mice after 5 weeks (*n* = 5 for each group). **(A)** Illustration showing obesity induction and amelioration after treatment. **(B)** Scanning electron micrograph image of *A. muciniphila*-derived EVs. **(C)** Body weight gain per mouse. **(D)** Average daily food intake. **(E)** Epidydimal adipose weight (EAT). **(F)** Histopathology image. Scale bar is 50 μm. **(G)** Total cholesterol levels. **(H)** Triglyceride levels and **(I)** glucose levels. ^∗^*P* < 0.05 and ^∗∗^*P* < 0.01 were considered statistically significant, respectively. ND, normal diet + PBS; NA.m, normal diet + *A. muciniphila* (10^9^ CFU); NEV, normal diet + EVs (10 μg protein); HFD, high-fat diet + PBS; HA.m, high-fat diet + *A. muciniphila* (10^9^ CFU); HEV, high-fat diet + EVs (10 μg protein).

Thirty male C57BL/6 mice were purchased from Pasteur Institute of Karaj (Iran), maintained in equal conditions (12 h light, 22–23°C, and 40% humidity) with *ad libitum* access to food and autoclaved water. After 1 week of acclimation with standard normal diet (ND) (A03, safe diet, France), 8-week-old mice were randomly divided into two groups, and each group was divided into three subgroups as follows:

The first group was fed HFD (260 HF 60% energy from butter, safe diet, France) for 3 months. After weight gain, treatment for 5 weeks along with HFD (Figure 1A):

(1) HFD + 200 μl PBS (HPBS)
(2) HFD + 10^9^ CFU/200 μl alive *A. muciniphila* (HA.m)
(3) HFD + 10 μg protein/200 μl EVs (HEV)

The second group was fed normal diet (A03) under the above conditions (Figure 1A):

(1) ND + 200 μl PBS (NPBS)
(2) ND + 10^9^ CFU/200 μl alive *A. muciniphila* (NA.m)
(3) ND + 10 μg protein/200 μl EVs (NEV)

Body weight, average food, and water intake were measured once a week. Note that at the end of the experiment, HFD-fed mice exhibiting aggressive behavior and resistant to obesity were excluded from our study. Before and after oral gavage, blood was collected *via* tail vein and stored at –80°C until biochemical analysis. At the end of treatment, all of the mice were sacrificed by cervical dislocation, and EAT and colon samples were snap frozen with liquid nitrogen and stored at –80°C. Moreover, the tissues are saved for histopathology analysis. We followed the institute guidelines regarding the care and use of laboratory animals. The study protocol was approved by the Animal Experiment Committee of Pasteur Institute of Iran.

### Computation of Selected Gene Expression

Gene expression datasets were downloaded from the National Center for Biotechnology Information gene expression omnibus (GEO). Our inclusion criteria to obtain relevant datasets were as follows: (a) studies qualifying as HFD experiments; (b) mouse samples for the experimental setups; (c) the number of samples for each group ND and HFD must be more than one; (d) using epididymal adipose tissue; and (e) survey the expression of all selected genes. A total of nine datasets (Table 1) that qualified for our study were obtained.

**TABLE 1:** List of datasets used in meta-analysis.

**GEO series ID**	**GEO platform ID**	**No. of ND sample**	**No. of HFD sample**	**HFD (% of energy from fat)**	**Sample**	**Submission date**
GSE65557	GPL6246 [(MoGene-1_0-st) Affymetrix Mouse Gene 1.0 ST Array (transcript (gene) version)]	3	3	60%	EAT	2015
GSE44653		3	3	42%	EAT	2013
GSE28440		3	3	45%	EAT	2011
GSE100388	GPL1261 [(Mouse430_2) Affymetrix Mouse Genome 430 2.0 Array]	5	5	39.9%	EAT	2017
GSE71367		9	9	39.9%	EAT	2015
GSE19954		2	2	30%	EAT	2010
GSE36033	GPL11533 [(MoGene-1_1-st) Affymetrix Mouse Gene 1.1 ST Array (transcript (gene) version)]	3	3	60%	EAT	2012
GSE39549	GPL6887 (Illumina MouseWG-6 v2.0 expression beadchip)	3	3	39.2%	EAT	2012

All analyses were undertaken using the R (version 3.5.2) statistical computing environment and Bioconductor (version 3.9). The individual datasets were normalized followed by log2 transformation before meta-analysis. We mapped the probeset identifiers in each gene expression dataset into their corresponding gene symbol using an annotation table available with the respective data at GEO. Multiple probeset identifiers can be mapped to the same gene symbol, and we removed the duplicity by using the means factor of respective entry. We performed data merging to correct for batch effects using the ComBat function of the SVA package (Leek et al., 2019). Then, we surveyed the expression of all selected genes across the nine datasets.

### Biochemical Parameters Analysis

After blood collection, fasting blood glucose (Glc), total cholesterol (TC), and triglyceride (TG) concentrations were measured in the plasma using a commercial kit (Bioclin-Quibasa, Belo Horizonte, MG, Brazil).

### Histological Evaluation

At first, colon and EAT were immersed in 10% neutral buffered formalin, dehydrated, and embedded in paraffin. The tissue sections were stained with hematoxylin and eosin (H&E). Microscopic observation of the histological slides was performed using a light microscope (Erben et al., 2014).

### Tissue RNA Isolation, cDNA Synthesis, and Real-Time PCR

Frozen colon and EAT were homogenized in 1 ml of Trizol (cat # BS410, Bio Basic, Canada) using a Precellys 24 homogenizer, and then total RNA was extracted according to the manufacturer’s instructions. For genomic DNA removal, RNA was treated with DNase I (Qiagen) and then cDNA was synthesized using PrimeScript RT Reagent Kit (Takara). Real-time PCR was performed using SYBR Premix Ex Taq II (Takara). A sequence of primers used in this study is shown in Table 2.

**TABLE 2:** Sequence of primers used in qPCR in cell line and mice.

**Primer name**	**Forward primer**	**Reverse primer**	**Product size (bp)**
*m-RPL-19*	TCAGCCACAACATTCTCA	GCACCTCCAACAGTAAGT	138
*m-TLR-2*	TCCTGCGAACTCCTATCC	CCTGGTGACATTCCAAGAC	151
*m-TLR-4*	GCCTTTCAGGGAATTAAGCTCC	GATCAACCGATGGACGTGTAAA	114
*m-ZO-1*	GCCGCTAAGAGCACAGCAA	TCCCCACTCTGAAAATGAGGA	134
*m-CLDN-1*	TCTGCCACTTCTCACTTCCA	GCCTATACCCTTGCTCTCTGT	95
*m-CLDN-2*	CAACTGGTGGGCTACATCCTA	CCCTTGGAAAAGCCAACCG	128
*m-OCLDN*	TTGAAAGTCCACCTCCTTACAGA	CCGGATAAAAAGAGTACGCTGG	129
*m-IL-10*	GCACTACCAAAGCCACAAG	AGTAAGAGCAGGCAGCATAG	85
*m-TNF-*α	AACAACTACTCAGAAACACAAG	GCAGAACTCAGGAATGGA	130
*m-ANGPTL4*	ACTGTGAGATGACTTCAGATGG	ATTGGCTTCCTCGGTTCC	174
*m-HPRT*	TCAGTCAACGGGGGACATAAA	GGGGCTGTACTGCTTAACCAG	142
*m-PPAR-*α	CACTTGCTCACTACTGTCCTT	GATGCTGGTATCGGCTCAA	110
*m-PPAR-*γ	GGTGCTCCAGAAGATGACAGA	TCAGCGGGTGGGACTTTC	154
*m-TGF-*β*1*	AATTCCTGGCGTTACCTT	TGTATTCCGTCTCCTTGG	116
*m-IL-6*	TCCATCCAGTTGCCTTCT	TAAGCCTCCGACTTGTGAA	137
*GAPDH*	GGAGCGAGATCCCTCCAAAAT	GGCTGTTGTCATACTTCTCATGG	197
*TLR-2*	TTATCCAGCACACGAATACACAG	AGGCATCTGGTAGAGTCATCAA	160
*TLR-4*	AGACCTGTCCCTGAACCCTAT	CGATGGACTTCTAAACCAGCCA	147
*ZO-1*	CAACATACAGTGACGCTTCACA	CACTATTGACGTTTCCCCACTC	105
*OCLDN*	AAGAGTTGACAGTCCCATGGCATAC	ATCCACAGGCGAAGTTAATGGAAG	133
*CLDN-1*	GCATGAAGTGTATGAAGTGCTTGG	CGATTCTATTGCCATACCATGCTG	132
*ANGPTL4*	ATGCCCAGTACGAACATTTCC	CTGAGTCAAGGGTGCTAAAGC	135

*m, mice.*

### Cell Culture Conditions

The Caco-2 (ATCC^®^ HTB-37) was cultured at 37°C in 5% CO_2_ in Dulbecco’s modified eagle’s medium (DMEM) (Gibco, United Kingdom), supplemented with 10% heat-inactivated fetal bovine serum (FBS, Gibco), and 1% penicillin–streptomycin (Gibco) in six-well plates (Sorfa, China). The medium was changed every 2–3 days. *Mycoplasma* spp. contamination was assessed using PCR method.

### Cell Culture Treatment

After 21 days, Caco-2 monolayer was infected with *A. muciniphila* at multiplicity of infection (MOI) ratios of 100 (100 bacteria per cell). Besides, 10 μg of EV was used for Caco-2 treatment. An equal volume of PBS was used as a control. Hence, cell viability was checked after treatments.

### RNA Extraction, cDNA Synthesis, and Quantitative Real-Time PCR

After 24 h, total RNA was extracted from treated cells using the RNeasy Plus Mini Kit (Qiagen, United States, Cat No./ID: 74134). cDNA synthesis was performed by PrimeScript RT Reagent Kit (Takara, Japan, Cat. # RR037A) according to the manufacturer’s instruction. Real-time PCR was performed by 2X SYBR Premix Ex Taq II (Tli RNase H, Plus Takara, Japan, Cat. #RR820L). A sequence of primers used in this study is shown in Table 2.

### Cytokine Assay

After 24 h post-treatment, both treated and untreated cell supernatants were collected and stored at –80°C. After thawing and prior to using, the supernatant was centrifuged for 10 min at 1000 g to remove any residual cells. The level of cytokines and chemokines (IL-1A, IL-1B, IL-2, IL-4, IL-6, IL-8, IL-10, IL-12, IL-17A, IFN-γ, TNF-α, and GM-CSF) was measured by Human Inflammatory Cytokines Multi-Analyte ELISA Array^TM^ Kits (Qiagen, Cat. no. MEH-004A) according to the manufacturer’s instructions. This assay was performed in duplicate.

### Statistical Analysis

The ΔΔCT method was used for the relative gene expression analysis, and *GAPDH* in cell line and *RPL-19* in colon and *HPRT* in adipose tissue of mice were used as an internal control. GraphPad Prism 8.0 (GraphPad Software Inc., CA, United States) was used for cycle threshold (CT) values analysis to calculate changes in gene expression and cytokine concentration comparison. A *P* value of less than 0.05 was considered statistically significant.

## Results

### Morphology and Size Range of EVs

The extracted EVs from *A. muciniphila* were evaluated by SEM; the results showed a spherical shape and a range of 40 to 150 nm in size (Figure 1B).

### *A. muciniphila* and Its EVs Reduced Food Intake and Body and Adipose Weight Gain

High-fat diet-induced obese mice were used in this experiment to evaluate the role of *A. muciniphila* and its EVs on weight control. HFD-fed mice showed increase diet-induced body weight after 3 months. Obese mice were gavaged by *A. muciniphila-*derived EVs and showed low level of body weight gain and significant reduction in food intake (Figures 1C,D). Hence, feeding obese mice with this bacterium caused body and EAT weight loss, but lower effect on body weight and adipose weight was observed compared to its EVs (Figures 1C,E). Interestingly, both of them significantly influence body weight in ND mice, whereas food intake did not change significantly (Figure 1D). The HFD group included the largest adipocytes compared to other groups (Figure 1F). In HFD-fed mice, administration of this bacterium and EVs considerably decreased the adipocyte size while the effects of EVs were more noticeable (Figure 1F). The smallest adipocytes were found in the ND group that received *A. muciniphila* and its EVs, compared to that in other groups (Figure 1F). Overall, the adipocyte size and infiltration were enhanced after feeding HFD whereas administration with this bacterium and EVs reduced them (Figure 1F).

The extracellular vesicles corrected HFD-induced hypercholesterolemia with significantly lower plasma TC (Figure 1G). After EVs administration, TG levels did not change (Figure 1H). Moreover, treatment with EVs displayed significantly lower plasma glucose in both groups (Figure 1I). Obese mice treated with *A. muciniphila* displayed significantly lower plasma TG and glucose concentrations (Figures 1H,I). In the normal group, the bacterium treatment elicited significantly decreasing plasma TG and glucose levels compared to the control (PBS) group (Figures 1H,I).

### Different Genes Involved in Inflammation and FA Oxidation Change in the Adipose Tissues of HFD-Fed Mice

**FIGURE 2:**
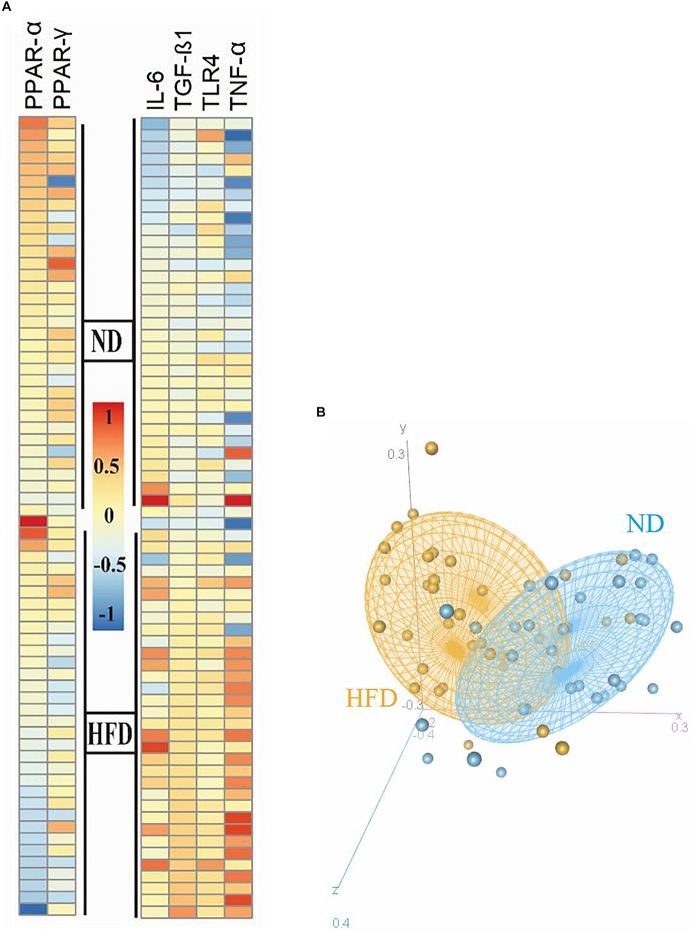
Heatmap and PCA correlation show different genes involved in inflammation and FA oxidation in the epididymal adipose tissue between HFD and ND mice. **(A)** Heatmap plot revealed inflammatory genes (*TNF-*α, *IL-6*, and *TLR-4*) and *TGF-*β were expressed at significantly higher levels in the obese than in the normal group. **(B)** Regulator genes involved in FA oxidation and inflammation (*PPAR-*γ and *PPAR-*α) were downregulated in HFD- compared to ND-fed mice. PCA plot showed that the ND group was clustered relatively from the HFD group. The HFD and ND groups are indicated by orange and blue colors, respectively.

After meta-analyzing, we found that the expression of these genes in EAT are closely related to obesity and were significantly differentially expressed with *P* value < 0.05 by the Mann–Whitney method. As it is shown in the heatmap plot (Figure 2A), inflammatory mediators (i.e., TNF-α and IL-6), TLR-4, and TGF-β were expressed at significantly higher levels in the obese than in the normal group (*P* value TLR4 and IL-6 = 0.0004, TNF-α and TGF-β < 0.0001), moreover regulator genes involved in FA oxidation and inflammation were downregulated in HFD- compared to ND-fed mice (*P* value PPAR-γ = 0.01 and PPAR-α = 0.001). Principal component analysis (PCA) was applied for the selected gene between all 34 HFD and 34 ND samples from nine datasets. PCA (Figure 2B) showed that the ND group (indicated by blue color) was clustered relatively from the HFD group (indicated by orange color). Since these genes showed similar and significant trends in all datasets and also play a key role in adiposity and inflammation, these genes were selected to study the effect of *A. muciniphila* and its EVs on obesity-related genes.

### Treatment With *A. muciniphila* and Its EVs Was Correlated With the Regulation of Gene Expression Involved in FA Oxidation and Energy Metabolism of Adipose Tissues

**FIGURE 3:**
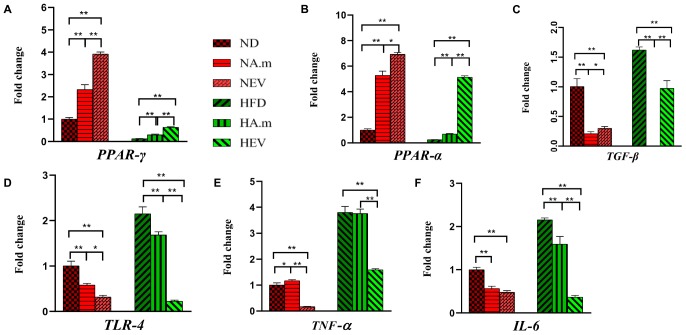
The effect of *A. muciniphila* and its EVs on mRNA expression of genes in epidydimal adipose tissue in C57bl/6 mice. Relative mRNA expression of **(A)** *PPAR*-γ, **(B)** *PPAR-*α, **(C)** *TGF-*β, **(D)** *TLR-4*, **(E)** *TNF-*α, and **(F)** *IL-6.* Data are normalized using *HPRT* as control gene. ^∗^*P* < 0.05 and ^∗∗^*P* < 0.01 were considered statistically significant, respectively. ND, normal diet + PBS; NA.m, normal diet + *A. muciniphila* (10^9^ CFU); NEV, normal diet + EVs (10 μg protein); HFD, high-fat diet + PBS; HA.m, high-fat diet + *A. muciniphila* (10^9^ CFU); HEV, high-fat diet + EVs (10 μg protein).

To investigate the effect of *A. muciniphila* and its EVs on FA oxidation and energy metabolism in mice, expression of *PPAR-*α/γ and *TGF-*β in adipose tissue was evaluated by real-time PCR. In obese mice, *A. muciniphila* influenced fatty acid oxidation and energy metabolism, accompanied by increased expression of *PPAR-*α, and *PPAR*-γ in adipose tissue (Figures 3A,B). The EVs significantly induced overexpression of *PPAR-*α in EAT in obese groups in comparison to the bacterium (Figure 3B). Both treatments enhanced expression of *PPAR-*α and *PPAR*-γ in normal mice, and the EV effects were more noticeable (Figures 3A,B). In obese mice, a significant rise in levels of *TGF-*β expression in EAT was observed; in contrast, in normal mice being treated with the bacterium, EVs reduced this expression (Figure 3C). The bacterium induced significantly more reduction in levels of *TGF-*β expression in EAT of obese and normal groups, compared to EVs (Figure 3C).

### Adipose Inflammation Alleviated After Oral Administration of *A. muciniphila* and Its EVs in Obese Mice

Since obesity is associated with a low-grade inflammatory state in adipose tissues, the role of *A. muciniphila* and its EVs on expression of adipose inflammatory genes was assessed in HFD-induced obese mice. Administration of *A. muciniphila* reduced the mRNA expression of *TLR-4* and *IL-6* genes in EAT in both groups, but it did not affect *TNF-*α expression in obese mice, while there was an increase in this gene in normal mice (Figures 3D–F). However, EVs induced more reduction in inflammatory cytokines (*TNF-*α and *IL-6*) and *TLR-4* expression in HFD mice (Figures 3D–F). In normal mice, EVs had more reduction in *TNF-*α and *TLR-4* expression (Figures 3D,E), compared to the bacterium.

### Administration of *A. muciniphila* and Its EVs Ameliorated HFD-Induced Intestinal Barrier Dysfunction in Obese Mice

**FIGURE 4:**
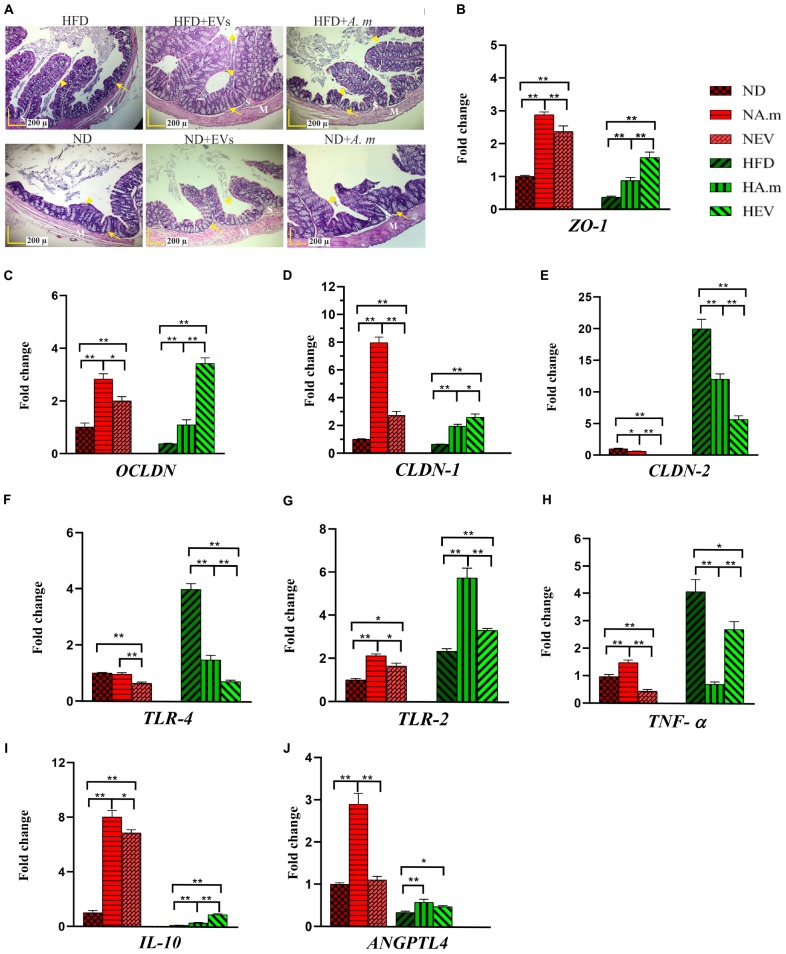
The assessment of *A. muciniphila* and its EVs effect on obesity-related genes in the colon of HFD- and ND-fed mice. Both mice administrated to this bacterium and EVs for 5 weeks. **(A)** Histopathology image. M, tunica muscularis; S, submucosa layer. Crypt depth (arrows). Mucous thickness (arrowheads). Scale bar is 200 μm. Expression of **(B)** *ZO-1*, **(C)** *OCLDN*, **(D)** *CLDN-1*, **(E)** *CLDN-2*, **(F)** *TLR-4*, **(G)** *TLR-2*, **(H)** *TNF-*α, **(I)** *IL-10*, and **(J)** *ANGPTL4*. Data are normalized using *RPL-19* as control gene. ^∗^*P* < 0.05 and ^∗∗^*P* < 0.01 were considered statistically significant, respectively. ND, normal diet + PBS; NA.m, normal diet + *A. muciniphila* (10^9^ CFU); NEV, normal diet + EVs (10 μg protein); HFD, high-fat diet + PBS; HA.m, high-fat diet + *A. muciniphila* (10^9^ CFU); HEV, high fat-diet + EVs (10 μg protein).

**FIGURE 5:**
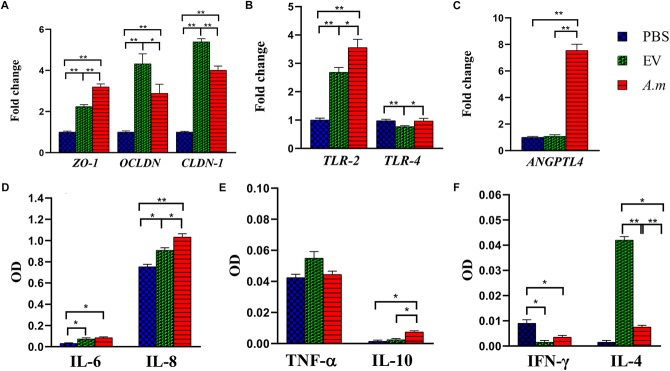
The effect of *A. muciniphila* and its EVs on the study’s genes and cytokine secretion in the Caco-2 cell line. Caco-2 monolayers were treated with *A. muciniphila* (MOI_100_) and EV concentration (10 μg) for 24 h. The expression of genes: **(A)** *ZO-1, OCLDN*, and *CLDN-1*; **(B)** *TLR-2* and *TLR-4*; and **(C)** *ANGPTL4*. The levels of cytokines: **(D)** IL-6 and IL-8; **(E)** TNF-α and IL-10; and **(F)** IFN-γ and IL-4. Expression data are normalized using *GAPDH* as control gene. ^∗^*P* < 0.05 and ^∗∗^*P* < 0.01 were considered statistically significant, respectively.

The colon is the first site affected by HFD, and dysbiosis induces different obesity pathways. Therefore, we investigated the effects of *A. muciniphila* and its EVs on pathology of colon tissues. Crypt depth and thickness of the mucous layer of the colon showed a considerable decrease in the HFD group, compared to that in the control group. Furthermore, mild infiltration of inflammatory cells in the lamina propria of the colon was present after HFD. On the contrary, no inflammatory reaction was present in other groups. An increase in crypt depth and mucosal thickness of colon tissue was observed in groups that received *A. muciniphila* and its EVs, compared to that in the HFD group (a). In the normal group, both administration of the bacterium and its EVs showed an increase in mucosal thickness (Figure 4A). Since an increase in the intestinal mucosal permeability occurs in obesity, HFD-induced obese mice that disrupted the intestinal barrier were selected. In obese mice, the EVs significantly reduced gut barrier permeability. These observations were accompanied by upregulated tight junction expression (*ZO-1*, *OCLDN*, and *CLDN-1*) and downregulated *CLDN-2* in the colon of mice treated with EVs (Figures 4B–E). There was an increase in mRNA level of tight junction in response to being treated with *A. muciniphila* in obese mice, while EVs had better effect. In normal mice, the bacterium effects were more noticeable (Figures 4B–E). Also, to determine whether *A. muciniphila* and its EVs have a direct effect on the intestinal epithelial cells or indirectly play a role by making changes in the composition of gut microbiota, Caco-2 cell lines were used. In terms of cell viability, more than 98% of the cells survived after treatment with the bacterium, and its EVs (data not shown). After treatment, the bacterium and EVs strongly increased intestinal integrity by tight junction genes’ upregulation in the cell line (Figure 5A).

### *A. muciniphila* and Its EVs Regulated Inflammation and Energy Homeostasis in the Colon of Obese Mice

High-fat diet seems to cause disruption of the intestinal barrier and consequently leads to inflammation. Therefore, the anti-inflammatory effect of *A. muciniphila* and its EVs by TLRs was evaluated in HFD-induced mice. After EV administration, the expression of *TLR-4* reduced more in obese mice when compared with this bacterium (Figure 4F). In comparison with *A*. *muciniphila*, EVs induced significantly lower *TLR-2* expression in both mice’s colon (Figure 4G). In the Caco-2 cell line, this bacterium did not stimulate *TLR-4* while it induced *TLR-2* expression. Similar to *in vivo*, EVs induced upregulation in expression of *TLR-2* and downregulation in expression of *TLR-4* in the cell line (Figure 5B). Both this bacterium and EVs induced intestinal immune homeostasis through regulated ratio of pro- and anti-inflammatory cytokine expression in obese mice’s colon (Figures 4H,I). In normal mice, a slight increase of pro-inflammatory cytokine *TNF-*α expression was observed after being gavaged by the bacterium (Figure 4H). Conversely, the EVs downregulated significantly the expression of this cytokine. Also, significant anti-inflammatory cytokine *IL-10* upregulation was seen after both administration in normal mice (Figure 4I). *A*. *muciniphila* and its EVs induced both pro- and anti-inflammatory cytokine secretion, while EVs induced significantly lower levels of IL-8 and the levels of IL-4 increased compared with *A*. *muciniphila* in the Caco-2 cell line (Figures 5D,F). *A*. *muciniphila* had a significant effect on an increase in the level of IL-10, compared to EVs (Figure 5E). In addition, the increase in TNF-α level following both treatments was not significant (Figure 5E). Both treatments significantly reduced the level of IFN-γ in comparison with the control group (Figure 5F).

To investigate the role of *A. muciniphila* and its EVs on fat storage, gene expression of Angiopoietin-like 4 (ANGPTL4), which plays a key role in regulatory pathways involving energy and lipid metabolism under HFD, was evaluated in colon of mice. Treatment of *A. muciniphila* and its EVs influences improvement of lipid and energy metabolism in obese mice. These observations were accompanied by increased expression of intestinal ANGPTL4 expression in mice treated with this bacterium (Figure 4J). Moreover, administration of EVs showed slight effects on ANGPTL4 expression in obese mice (Figure 4J). Additionally, treatment with *A. muciniphila* in normal mice induced a high mRNA level of ANGPTL4 in the colon (Figure 4J), whereas EVs were not effective in Angptl4 upregulation, similar to the *in vitro* study (Figure 5C).

## Discussion

Obesity is characterized by weight gain, fat accumulation, disruption of metabolic and energy homeostasis, and low-grade inflammation (Choe et al., 2016; WHO, 2016). Multiple studies have confirmed the effects of probiotics on obesity, as they improve metabolic parameters and reduce body weight by changing the microbiota composition (Schneeberger et al., 2015; Ejtahed et al., 2019). Recent evidences show that OMV-released probiotic and commensal bacteria can internalize intestinal epithelial cells and play a crucial role in the modulation of various pathways (Alvarez et al., 2016; Cañas et al., 2016; Fábrega et al., 2016). Our findings showed that *A. muciniphila*-derived EVs alleviated more body and fat weight gain in obese mice after 5 weeks, compared to the bacterium. Both treatments had effects on the weight of normal mice, while the bacterium had greater effects on EAT weight. In addition, blood glucose and cholesterol levels reduced following treatment with EVs in obese mice.

In comparison with the control group, administration of *A. muciniphila* induced significantly lower levels of glucose and TG in obese mice. In normal mice, the bacterium had more significant effects on TG, compared with EVs. These results may indicate the potential role of the bacterium and its EVs in obesity treatment. Similarly, a recent study revealed that the abundance of *A. muciniphila* decreased in HFD-fed mice, which is strongly correlated with weight gain, increased inflammation, increased expression of lipid-metabolism genes in adipose tissues, and increased level of blood markers (e.g., lipid and glucose) in obese mice (Schneeberger et al., 2015). Accumulating evidence shows that probiotic administration leads to a reduction in body weight gain, adipose weight, and obesity biomarkers (e.g., glucose and plasma lipid) in HFD-fed mice (Rather et al., 2014; Parker et al., 2017). In addition, the effect of probiotics on the improvement of metabolism was confirmed in normal mice (Zhao et al., 2017).

Several studies indicate that HFD induces an increase in adipocyte size, macrophage infiltration, and secretion of inflammatory mediators associated with obesity pathogenesis (Blüher, 2016). Our meta-analysis, consistent with previous studies, showed that the levels of PPAR-γ and PPAR-α decreased due to obesity, while TGF-β level and inflammation increased in adipose tissues. PPARs, as lipid sensors, regulate the host metabolism by affecting the genes involved in fat storage and energy homeostasis and modulate inflammatory responses; accordingly, they are regarded as the link between lipid signaling and inflammation (Wahli and Michalik, 2012). Previous findings suggest that PPAR-γ contributes to the regulation of FA metabolism and alleviation of inflammation and fibrosis (Huang and Glass, 2010). Moreover, increased activation of PPAR-α in adipocytes induces FA oxidation, decreases food intake, increases energy expenditure, and regulates inflammatory responses (Goto et al., 2011; Allen and Bradford, 2012; Gross et al., 2017). A recent study revealed that activation of both PPAR-α and PPAR-γ by synthetic agonists might have greater effects on inflammation and obesity amelioration (Feng et al., 2016). Our findings showed that administration of *A. muciniphila* and its EVs triggers an increase in PPAR-α and PPAR-γ mRNA content of EAT in both groups, which is indicative of their influence on FA oxidation and energy metabolism. Notably, EVs had a greater effect on the expression of PPARs in EAT in comparison to the bacterium.

In a previous study, administration of *Lactobacillus gasseri* BNR17 showed that alleviation of white adipose tissue weight gain increases FA oxidation by changing *PPAR-*α expression (Kang J.-H. et al., 2013). An increase in the level of TGF-β in adipose tissues influences the production of inflammatory mediators, regulation of energy homeostasis, fat mass expansion, and promotion of collagen deposition, which are associated with obesity in animals and humans (Samad et al., 1997; Fain et al., 2005; Lin et al., 2009; Yadav et al., 2011; Sousa-Pinto et al., 2016). Yadav et al. (2011) showed that neutralization of TGF-β with antibodies has protective effects on diabetes and obesity by improving metabolism. We found that HFD causes an increase in *TGF-*β mRNA content of EAT, compared with the normal diet; in fact, the mRNA level decreased after both administrations in mice. These findings may indicate the potential effect of this bacterium and its EVs on regulating energy metabolism and counteracting obesity.

A large number of studies have reported a correlation between obesity amelioration and PPARs, which directly affect the reduction of inflammatory cytokines or indirectly influence lipid metabolism in adipose tissues (Stienstra et al., 2007; Wahli and Michalik, 2012). PPAR-α and PPAR-γ are capable of reducing the expression of pro-inflammatory cytokines through influencing adipose size and reversing macrophage infiltration, respectively (Tsuchida et al., 2005; Stienstra et al., 2007). On the other hand, activation of TLR-4 in adipose tissues occurs through interactions with gut microbiota LPS or adipocyte lipolysis-released free fatty acids, which can induce the secretion of inflammatory adipocytokines (i.e., IL-6 and TNF) in obesity (Cani et al., 2007; Gross et al., 2017).

Besides the impact on PPARs, we found that EVs have more significant anti-inflammatory effects on the EAT of obese mice, compared with the bacterium itself. These effects are accompanied by a decrease in the expression of *TLR-4* and pro-inflammatory cytokines, *TNF-*α and *IL-6*, in EAT.

In addition, both treatments had positive effects on normal mice. Several animal studies have shown that probiotic treatments downregulate pro-inflammatory cytokine genes and upregulate the expression of FA oxidation-related genes in white adipose tissues (Núñez et al., 2015; Qiao et al., 2015). Collectively, our results showed that both treatments could ameliorate obesity and reverse the obesity effects. These results possibly represent that nano-sized EVs can enter the bloodstream and access other tissues such as adipose tissue as well as regulate a variety of cellular and molecular pathways.

Correlation of dysbiosis with obesity is indicated in several animal and human studies (Cani et al., 2008; Armougom et al., 2009; Turnbaugh et al., 2009; Fei and Zhao, 2013; Schneeberger et al., 2015). It is known that obesity increases intestinal permeability, caused by switching of tight junction proteins (Kim et al., 2012). Therefore, the level of these proteins such as zonula occludens-1 (ZO-1), occludin (OCLDN), and claudin-1 (CLDN-1) reduces, while *CLDN-2* expression in permeable epithelial cell increases (Everard et al., 2013; Ahmad et al., 2017). Our findings showed that HFD-induced colon barrier dysfunction improves significantly after administration of EVs by switching tight junction protein expression in obese mice. Moreover, *A. muciniphila* and its EVs upregulated the expression of tight junction proteins not only in Caco-2 cells, but also in mice.

Additionally, we found a significant increase in *CLDN-2* expression among HFD-fed mice, while EVs had a considerable effect on *CLDN-2* downregulation in obese mice. In this regard, a previous study showed that *A. muciniphila* could regulate tight junction protein expression and strengthen the intestinal barrier in obese mice by affecting *TLR-2* expression (Plovier et al., 2017). Chelakkot et al. (2018) demonstrated weight loss in HFD-fed mice after treatment with *A. muciniphila*-derived EVs, which also diminished the HFD-induced gut permeability. Moreover, a recent study showed that *A. muciniphila* EVs have more beneficial effects on the mucosal integrity and weight gain of colitis mice, compared with the bacterium (Kang C.-S. et al., 2013). Other animal studies have shown that oral administration of probiotics improves the gut barrier integrity and metabolic disorders (Everard et al., 2013; Plovier et al., 2017; Le Barz et al., 2018). In line with the abovementioned findings, we showed that probiotics and their bioactive factors reinforce the epithelial barrier integrity through upregulation of several tight junction-related genes. An increase in the gut permeability stimulates the immune cells in the lamina propria by pathogenic and beneficial bacteria (i.e., LPS) and ultimately leads to inflammation. Probiotic, as a potential therapeutic option to target obesity, interacts with TLRs on intestinal epithelial or immune cells and stimulates the secretion of various cytokines in the gut, ultimately leading to mucosal immune homeostasis (Van Baarlen et al., 2013).

Since *Akkermansia muciniphila* is closer to the host epithelial cells, it exhibits significant immune regulatory responses. In our study, the cell line, after exposure to *A. muciniphila*, showed activated cells expressing *TLR-2*, but no change in *TLR-4* expression was found, similar to normal mice. Generally, TLR-4 is the best marker for identifying Gram-negative LPS, while commensal bacterial LPS are different from pathogenic bacteria, which stimulate TLR-2 instead of TLR-4 and cause immune system tolerance (Alhawi et al., 2009). In this regard, an *in vitro* study, comparing the effects of *A. muciniphila* with *E. coli* LPS on HT29 cells, showed that *A. muciniphila* LPS are distinct from *E. coli* LPS (Reunanen et al., 2015). Conversely, another study demonstrated that *A. muciniphila* is a strong inducer of TLR-4 activation in HEK-Blue cells (Ottman et al., 2017). Differences in TLR-4 stimulation may be due to variations in cell type and culture medium.

Recent studies have revealed that gut microbiota possibly communicates with the host by secreting EVs and mediates immune signaling pathways by interacting with TLRs (Shen et al., 2012; Fábrega et al., 2016). Our findings showed that treatment with *A. muciniphila*-derived EVs activated *TLR-2* expression, while reducing TLR-4 expression in the cell line and mice. In the present study, treatment with *A. muciniphila* in obese mice induced *TLR-2* upregulation and *TLR-4* downregulation. We found that the bacterium induces higher levels of pro-inflammatory cytokines in the cell line, compared with EVs. Similarly, another study on the same cell line showed that *A. muciniphila* stimulates IL-8 secretion, and the concentration of IL-8 was significantly lower than that reported in *E. coli* treatment (Reunanen et al., 2015). Moreover, another study reported that *A. muciniphila* EVs induce IL-6 secretion in CT26 cells. However, before treatment with *E. coli*-derived EVs, pretreatment with *A. muciniphila* EVs indicated the anti-inflammatory effects of these EVs (Kang C.-S. et al., 2013). Previous studies show that probiotics can modulate immune homeostasis by inducing pro- and anti-inflammatory cytokines in the cell line (Ottman et al., 2017; Rabiei et al., 2019). Following the administration of *A. muciniphila* in our study, high expression levels of pro-, and anti-inflammatory cytokines were observed in comparison with EV treatment in normal mice. These observations, which are in line with recent research, may show that induction of pro-inflammatory cytokine secretion by this bacterium in a normal intestine can play a key role in the immune system preparation for confronting bacterial pathogens and developing intestinal tolerance to commensal microbiota (Ottman et al., 2017).

On the other hand, gavage of obese mice with *A. muciniphila* and its EVs showed that both treatments could ameliorate intestinal inflammation. In addition, both *A. muciniphila* and its EVs induced anti-inflammatory cytokine expression in obese mice by affecting TLR-2 activation. Multiple studies have demonstrated that probiotics can ameliorate inflammation, caused by TLR-2-dependent induction of IL-10 secretion (Macho Fernandez et al., 2011; Jeon et al., 2012; Kaji et al., 2018). Overall, our findings, in line with previous studies, revealed the anti-inflammatory properties of *A. muciniphila* and its secretory vesicles in inflammatory diseases, especially obesity.

The gut microbiota can influence the energy balance, as well as weight control in the host through various mechanisms, including intestinal ANGPTL4, which diminishes lipogenesis, fat storage, and finally energy balance in the host (Jacouton et al., 2015; Tabasi et al., 2019). Moreover, it increases the plasma TG level and reduces inflammation (Galaup et al., 2012). Angptl4 is considered a link between the gut microbiota and obesity, preventing FA-induced inflammation (Lichtenstein et al., 2010). Since *ANGPTL4* expression reduces in obesity, use of probiotics for *ANGPTL4* upregulation may be the main target of HFD-induced obesity prevention or treatment (Zandbergen et al., 2006). In the current study, *A. muciniphila* induced the upregulation of *ANGPTL4* expression in both Caco-2 cell line and mice. A recent study also revealed that *A. muciniphila* metabolites, such as propionate, regulate *ANGPTL4* expression in intestinal organoids, and modulate lipid metabolism (Lukovac et al., 2014). Moreover, comparison of ANGPTL4 knockout (ANGPTL4^–/–^) and wild-type mice in an *in vivo* study showed that ANGPTL4^–/–^ mice do not counteract HFD-induced weight gain due to increased LPL activity (Bäckhed et al., 2004). In the present study, we found that bacterium-derived EVs are not effective in Angptl4 upregulation in the cell line and normal mice, which is in contrast with the slight change in Angptl4 expression in obese mice. Regarding *ANGPTL4* expression, it has been reported that treatment with live *Lactobacillus rhamnosus* CNCMI-4317 upregulates Angptl4 expression in HT29 cells, whereas the heat-inactivated bacterium and its supernatant do not affect Angptl4 regulation (Jacouton et al., 2015).

Our findings, in line with previous research, reveal that live probiotics probably affect the intestinal Angptl4 upregulation *via* surface molecules and prevent obesity (Jacouton et al., 2015). Moreover, the minor effect of EVs on the regulation of intestinal Angptl4 expression could be attributed to surface-exposed molecules in obese mice. These observations support the hypothesis that inflammation of the intestine, as the first site affected by HFD, can induce adipose inflammation and contribute to the progression of obesity (Kim et al., 2012). Therefore, early detection of intestinal inflammation and amelioration *via* probiotic treatment may have beneficial effects on the prevention of obesity.

**FIGURE 6:**
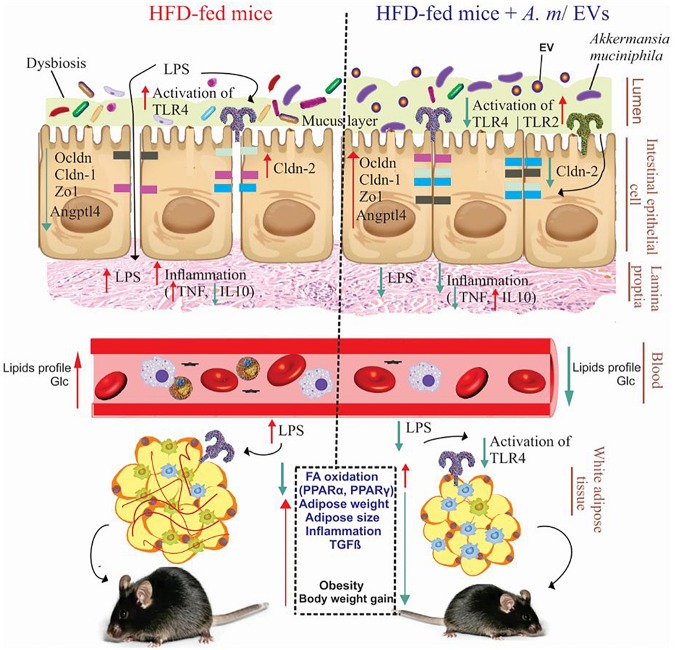
Administration of *A. muciniphila* and its EVs improves the intestinal and metabolic homeostasis in obese mice. Obesity is associated with the disruption of the intestinal barrier integrity, inflammation, and fat mass gain (left). Oral administration of *A. muciniphila* or its EVs increase the expression of tight junction proteins and *TLR-2* and reduce the expression of *TLR-4* and pro-inflammatory cytokines in the colon of obese mice (right). Also, treatment with *A. muciniphila* and its EVs affects FA oxidation, local inflammation genes, and fat-mass loss in the EAT of obese mice. HFD, high-fat diet; EVs, extracellular vesicles; *TLR*, toll-like receptor; LPS, lipopolysaccharide; PPAR, peroxisome proliferator-activated receptor; Zo-1, zonula occludens-1; Ocldn, occludin; Cldn, claudin; Angptl4, angiopoietin-like 4; TNF-α, tumor necrosis factor-α; IL-10, interleukin-10; TGF-β, transforming growth factor-β; and Glc, glucose.

In summary, HFD consumption can influence dysbiosis, inflammation, permeability of the mucosal barrier, accumulation of fat, and weight gain, and consequently lead to obesity. It was revealed that *A. muciniphila* and administration of its EVs could reverse adverse effects of obesity in obese mice (Figure 6). In fact, *A. muciniphila*-derived EVs showed more significant effects on adipose dysfunction, inflammation reduction, alleviation of intestinal permeability, and reversal of obesity effects compared to this bacterium. Therefore, these EVs may be considered as a possible target of HFD-induced obesity prevention or treatment. Finally, regarding the distinct effects of EV–host interaction on the treatment of obesity, further research is necessary to corroborate our findings.

## Conclusion

Despite molecular interactions between the intestinal microbiota, host energy balance, and inflammation, some obesity and clinical studies show that manipulation of gastrointestinal microbiota with probiotics can promote the pathophysiology of obesity. Our results indicated the crucial role of *A. muciniphila* and its EVs in health promotion and obesity treatment. Based on our findings and the leaky-gut hypothesis, *A. muciniphila*-derived EVs can be considered as more appropriate targets in new therapeutic strategies for obesity.

## Data Availability Statement

The datasets generated for this study are available on request to the corresponding author.

## Ethics Statement

The animal study was reviewed and approved by the Animal Experiment Committee of Pasteur Institute of Iran. Written informed consent was obtained from the owners for the participation of their animals in this study.

## Author Contributions

FA participated in experimental conception, RNA extraction, cDNA synthesis, real-time PCR, data interpretation, and manuscript writing. AS participated in bacterial culture, EVs extraction, gavage, and tissue homogenization. AB participated in cell culture, ELISA, and manuscript revision. HM participated in dissection of the mice, sampling of tissue, and histopathological evaluation. SR participated in gavage, tissue homogenization, and real-time PCR. AL performed the bioinformatics and meta-analyses. SH, SK, FV, and SS participated in manuscript revision. SH and RY helped in sampling of tissue. SS, FA, and SB participated in the design of the study. All authors read and approved the manuscript.

## Conflict of Interest

The authors declare that the research was conducted in the absence of any commercial or financial relationships that could be construed as a potential conflict of interest.

## References

[B1] Ahmad R. Rah B. Bastola D. Dhawan P. Singh A. B. (2017). Obesity-induces organ and tissue specific tight junction restructuring and barrier deregulation by claudin switching. *Sci. Rep.* 7:5125. 10.1038/s41598-017-04989-8 28698546 PMC5505957

[B2] Ahmadi Badi S. Moshiri A. Fateh A. Rahimi Jamnani F. Sarshar M. Vaziri F. (2017). Microbiota-derived extracellular vesicles as new systemic regulators. *Front. Microbiol.* 8:1610 10.3389/fmicb.2017.01610 PMC5573799 28883815

[B3] Alhawi M. Stewart J. Erridge C. Patrick S. Poxton I. R. (2009). *Bacteroides fragilis* signals through Toll-like receptor (TLR) 2 and not through TLR4. *J. Med. Microbiol.* 58 1015–1022. 10.1099/jmm.0.009936-0 19528164

[B4] Allen M. S. Bradford B. J. (2012). Control of food intake by metabolism of fuels: a comparison across species. *Proc. Nutr. Soc.* 71 401–409. 10.1017/S0029665112000572 22704548

[B5] Alvarez C.-S. Badia J. Bosch M. Giménez R. Baldomà L. (2016). Outer membrane vesicles and soluble factors released by probiotic *Escherichia coli* Nissle 1917 and commensal ECOR63 enhance barrier function by regulating expression of tight junction proteins in intestinal epithelial cells. *Front. Microbiol.* 7:1981. 10.3389/fmicb.2016.01981 28018313 PMC5156689

[B6] Armougom F. Henry M. Vialettes B. Raccah D. Raoult D. (2009). Monitoring bacterial community of human gut microbiota reveals an increase in *Lactobacillus* in obese patients and methanogens in anorexic patients. *PLoS One* 4:e7125. 10.1371/journal.pone.0007125 19774074 PMC2742902

[B7] Bäckhed F. Ding H. Wang T. Hooper L. V. Koh G. Y. Nagy A. (2004). The gut microbiota as an environmental factor that regulates fat storage. *Proc. Natl. Acad. Sci. U.S.A.* 101 15718–15723. 10.1073/pnas.0407076101 15505215 PMC524219

[B8] Behrouzi A. Vaziri F. Rad F. R. Amanzadeh A. Fateh A. Moshiri A. (2018). Comparative study of pathogenic and non-pathogenic *Escherichia coli* outer membrane vesicles and prediction of host-interactions with TLR signaling pathways. *BMC Res. Notes* 11:539. 10.1186/s13104-018-3648-3 30068381 PMC6071399

[B9] Blüher M. (2016). Adipose tissue inflammation: a cause or consequence of obesity-related insulin resistance? *Clin. Sci.* 130 1603–1614. 10.1042/CS20160005 27503945

[B10] Bouter K. E. van Raalte D. H. Groen A. K. Nieuwdorp M. (2017). Role of the gut microbiome in the pathogenesis of obesity and obesity-related metabolic dysfunction. *Gastroenterology* 152 1671–1678. 10.1053/j.gastro.2016.12.048 28192102

[B11] Cañas M. A. Giménez R. Fábrega M. J. Toloza L. Baldomà L. Badía J. (2016). Outer membrane vesicles from the probiotic *Escherichia coli* Nissle 1917 and the commensal ECOR12 enter intestinal epithelial cells *via* clathrin-dependent endocytosis and elicit differential effects on DNA damage. *PLoS One* 11:e0160374. 10.1371/journal.pone.0160374 27487076 PMC4972321

[B12] Cani P. D. Amar J. Iglesias M. A. Poggi M. Knauf C. Bastelica D. (2007). Metabolic endotoxemia initiates obesity and insulin resistance. *Diabetes* 56 1761–1772. 10.2337/db06-1491 17456850

[B13] Cani P. D. Bibiloni R. Knauf C. Waget A. Neyrinck A. M. Delzenne N. M. (2008). Changes in gut microbiota control metabolic endotoxemia-induced inflammation in high-fat diet–induced obesity and diabetes in mice. *Diabetes* 57 1470–1481. 10.2337/db07-1403 18305141

[B14] Cani P. D. de Vos W. M. (2017). Next-generation beneficial microbes: the case of *Akkermansia muciniphila*. *Front. Microbiol.* 8:1765. 10.3389/fmicb.2017.01765 29018410 PMC5614963

[B15] Chelakkot C. Choi Y. Kim D.-K. Park H. T. Ghim J. Kwon Y. (2018). *Akkermansia muciniphila*-derived extracellular vesicles influence gut permeability through the regulation of tight junctions. *Exp. Mol. Med.* 50:e450. 10.1038/emm.2017.282 29472701 PMC5903829

[B16] Choe S. S. Huh J. Y. Hwang I. J. Kim J. I. Kim J. B. (2016). Adipose tissue remodeling: its role in energy metabolism and metabolic disorders. *Front. Endocrinol.* 7:30 10.3389/fendo.2016.00030 PMC4829583 27148161

[B17] Dao M. C. Everard A. Aron-Wisnewsky J. Sokolovska N. Prifti E. Verger E. O. (2016). *Akkermansia muciniphila* and improved metabolic health during a dietary intervention in obesity: relationship with gut microbiome richness and ecology. *Gut* 65 426–436. 10.1136/gutjnl-2014-308778 26100928

[B18] David L. A. Maurice C. F. Carmody R. N. Gootenberg D. B. Button J. E. Wolfe B. E. (2014). Diet rapidly and reproducibly alters the human gut microbiome. *Nature* 505:559. 10.1038/nature12820 24336217 PMC3957428

[B19] Derrien M. Vaughan E. E. Plugge C. M. de Vos W. M. (2004). *Akkermansia municiphila* gen. *nov., sp. nov.*, a human intestinal mucin-degrading bacterium. *Int. J. Syst. Evol. Microbiol.* 54 1469–1476. 10.1099/ijs.0.02873-0 15388697

[B20] Ejtahed H.-S. Angoorani P. Soroush A.-R. Atlasi R. Hasani-Ranjbar S. Mortazavian A. M. (2019). Probiotics supplementation for the obesity management; a systematic review of animal studies and clinical trials. *J. Funct. Foods* 52 228–242. 10.1016/j.jff.2018.10.039

[B21] Ejtahed H.-S. Soroush A.-R. Angoorani P. Larijani B. Hasani-Ranjbar S. (2016). Gut microbiota as a target in the pathogenesis of metabolic disorders: a new approach to novel therapeutic agents. *Horm. Metab. Res.* 48 349–358. 10.1016/j.jff.2018.10.039 27203411

[B22] Emerging Risk Factors Collaboration (2011). Separate and combined associations of body-mass index and abdominal adiposity with cardiovascular disease: collaborative analysis of 58 prospective studies. *Lancet* 377 1085–1095. 10.1016/S0140-6736(11)60105-0 21397319 PMC3145074

[B23] Erben U. Loddenkemper C. Doerfel K. Spieckermann S. Haller D. Heimesaat M. M. (2014). A guide to histomorphological evaluation of intestinal inflammation in mouse models. *Int. J. Clin. Exp. Pathol.* 7:4557. 25197329 PMC4152019

[B24] Everard A. Belzer C. Geurts L. Ouwerkerk J. P. Druart C. Bindels L. B. (2013). Cross-talk between *Akkermansia muciniphila* and intestinal epithelium controls diet-induced obesity. *Proc. Natl. Acad. Sci. U.S.A.* 110 9066–9071. 10.1073/pnas.1219451110 23671105 PMC3670398

[B25] Fábrega M. J. Aguilera L. Giménez R. Varela E. Alexandra Cañas M. Antolín M. (2016). Activation of immune and defense responses in the intestinal mucosa by outer membrane vesicles of commensal and probiotic *Escherichia coli* strains. *Front. Microbiol.* 7:705. 10.3389/fmicb.2016.00705 27242727 PMC4863414

[B26] Fain J. N. Tichansky D. S. Madan A. K. (2005). Transforming growth factor β1 release by human adipose tissue is enhanced in obesity. *Metabolism* 54 1546–1551. 10.1016/j.metabol.2005.05.024 16253647

[B27] Fei N. Zhao L. (2013). An opportunistic pathogen isolated from the gut of an obese human causes obesity in germfree mice. *ISME J.* 7:880. 10.1038/ismej.2012.153 23235292 PMC3603399

[B28] Feng L. Luo H. Xu Z. Yang Z. Du G. Zhang Y. (2016). Bavachinin, as a novel natural pan-PPAR agonist, exhibits unique synergistic effects with synthetic PPAR-γ and PPAR-α agonists on carbohydrate and lipid metabolism in db/db and diet-induced obese mice. *Diabetologia* 59 1276–1286. 10.1007/s00125-016-3912-9 26983922

[B29] Galaup A. Gomez E. Souktani R. Durand M. Cazes A. Monnot C. (2012). Protection against myocardial infarction and no-reflow through preservation of vascular integrity by angiopoietin-like 4. *Circulation* 125 140–149. 10.1161/CIRCULATIONAHA.111.049072 22086875

[B30] Goto T. Lee J.-Y. Teraminami A. Kim Y.-I. Hirai S. Uemura T. (2011). Activation of peroxisome proliferator-activated receptor-alpha stimulates both differentiation and fatty acid oxidation in adipocytes. *J. Lipid Res.* 52 873–884. 10.1194/jlr.M011320 21324916 PMC3073464

[B31] Grant R. W. Dixit V. D. (2015). Adipose tissue as an immunological organ. *Obesity* 23 512–518. 10.1002/oby.21003 25612251 PMC4340740

[B32] Gross B. Pawlak M. Lefebvre P. Staels B. (2017). PPARs in obesity-induced T2DM, dyslipidaemia and NAFLD. *Nat. Rev. Endocrinol.* 13 36–49. 10.1038/nrendo.2016.135 27636730

[B33] Huang W. Glass C. K. (2010). Nuclear receptors and inflammation control: molecular mechanisms and pathophysiological relevance. *Arterioscler Thromb Vasc Bio* 30 1542–1549. 10.1161/ATVBAHA.109.191189 20631355 PMC2911147

[B34] Jacouton E. Mach N. Cadiou J. Lapaque N. Clément K. Doré J. (2015). *Lactobacillus rhamnosus* cncmi-4317 modulates fiaf/angptl4 in intestinal epithelial cells and circulating level in mice. *PLoS One* 10:e0138880. 10.1371/journal.pone.0138880 26439630 PMC4595210

[B35] Jeon S. G. Kayama H. Ueda Y. Takahashi T. Asahara T. Tsuji H. (2012). Probiotic *Bifidobacterium breve* induces IL-10-producing Tr1 cells in the colon. *PLoS Pathog.* 8:e1002714. 10.1371/journal.ppat.1002714 22693446 PMC3364948

[B36] Kaji R. Kiyoshima-Shibata J. Tsujibe S. Nanno M. Shida K. (2018). Probiotic induction of interleukin-10 and interleukin-12 production by macrophages is modulated by co-stimulation with microbial components. *J. Dairy Sci.* 101 2838–2841. 10.3168/jds.2017-13868 29397183

[B37] Kang C.-S. C.-S. Ban M. Choi E.-J. Moon H.-G. Jeon J.-S. Kim D.-K. (2013). Extracellular vesicles derived from gut microbiota, especially *Akkermansia muciniphila*, protect the progression of dextran sulfate sodium-induced colitis. *PLoS One* 8:e76520. 10.1371/journal.pone.0076520 24204633 PMC3811976

[B38] Kang J.-H. J.-H. Yun S.-I. Park M.-H. Park J.-H. Jeong S.-Y. Park H.-O. (2013). Anti-obesity effect of *Lactobacillus gasseri* BNR17 in high-sucrose diet-induced obese mice. *PLoS One* 8:e54617. 10.1371/journal.pone.0054617 23382926 PMC3559800

[B39] Kim K.-A. Gu W. Lee I.-A. Joh E.-H. Kim D.-H. (2012). High fat diet-induced gut microbiota exacerbates inflammation and obesity in mice *via the* TLR4 signaling pathway. *PLoS One* 7:e47713. 10.1371/journal.pone.0047713 23091640 PMC3473013

[B40] Lauby-Secretan B. Scoccianti C. Loomis D. Grosse Y. Bianchini F. Straif K. (2016). Body fatness and cancer—viewpoint of the IARC working group. *N. Engl. J. Med.* 375 794–798. 10.1056/NEJMsr1606602 27557308 PMC6754861

[B41] Le Barz M. Daniel N. Varin T. V. Naimi S. Demers-Mathieu V. Pilon G. (2018). In vivo screening of multiple bacterial strains identifies *Lactobacillus rhamnosus* Lb102 and *Bifidobacterium animalis* ssp. *lactis* Bf141 as probiotics that improve metabolic disorders in a mouse model of obesity. *FASEB J*. 33 4921–4935. 10.1096/fj.201801672R 30596521

[B42] Lee E. Y. Bang J. Y. Park G. W. Choi D. S. Kang J. S. Kim H. J. (2007). Global proteomic profiling of native outer membrane vesicles derived from *Escherichia coli*. *Proteomics* 7 3143–3153. 10.1002/pmic.200700196 17787032

[B43] Leek J. T. Johnson W. E. Parker H. S. Fertig E. J. Jaffe A. E. Storey J. D. (2019). *sva: Surrogate Variable Analysis. R package version 3.32.1*.

[B44] Lichtenstein L. Mattijssen F. de Wit N. J. Georgiadi A. Hooiveld G. J. van der Meer R. (2010). Angptl4 protects against severe proinflammatory effects of saturated fat by inhibiting fatty acid uptake into mesenteric lymph node macrophages. *Cell Metab.* 12 580–592. 10.1016/j.cmet.2010.11.002 21109191 PMC3387545

[B45] Lin Y. Nakachi K. Ito Y. Kikuchi S. Tamakoshi A. Yagyu K. (2009). Variations in serum transforming growth factor-β1 levels with gender, age and lifestyle factors of healthy Japanese adults. *Dis. Markers* 27 23–28. 10.3233/DMA-2009-0643 19822955 PMC3834674

[B46] Lukovac S. Belzer C. Pellis L. Keijser B. J. de Vos W. M. Montijn R. C. (2014). Differential modulation by *Akkermansia muciniphila* and *Faecalibacterium prausnitzii* of host peripheral lipid metabolism and histone acetylation in mouse gut organoids. *mBio* 5 e1438–14. 10.1128/mBio.01438-14 25118238 PMC4145684

[B47] Macho Fernandez E. Valenti V. Rockel C. Hermann C. Pot B. Boneca I. G. (2011). Anti-inflammatory capacity of selected lactobacilli in experimental colitis is driven by nod2-mediated recognition of a specific peptidoglycan-derived muropeptide. *Gut* 60 1050–1059. 10.1136/gut.2010.232918 21471573

[B48] Núñez I. N. Galdeano C. M. de LeBlanc A. D. M. Perdigón G. (2015). *Lactobacillus casei* CRL 431 administration decreases inflammatory cytokines in a diet-induced obese mouse model. *Nutrition* 31 1000–1007. 10.1016/j.nut.2015.02.006 26059375

[B49] Ottman N. Reunanen J. Meijerink M. Pietilä T. E. Kainulainen V. Klievink J. (2017). Pili-like proteins of *Akkermansia muciniphila* modulate host immune responses and gut barrier function. *PLoS One* 12:e0173004. 10.1371/journal.pone.0173004 28249045 PMC5332112

[B50] Parker E. P. K. Praharaj I. John J. Kaliappan S. P. Kampmann B. Kang G. (2017). Changes in the intestinal microbiota following the administration of azithromycin in a randomised placebo-controlled trial among infants in south India. *Sci. Rep.* 7:9168. 10.1038/s41598-017-06862-0 28835659 PMC5569098

[B51] Plovier H. Everard A. Druart C. Depommier C. Van Hul M. Geurts L. (2017). A purified membrane protein from *Akkermansia muciniphila* or the pasteurized bacterium improves metabolism in obese and diabetic mice. *Nat. Med.* 23 107–113. 10.1038/nm.4236 27892954

[B52] Qiao Y. Sun J. Xia S. Li L. Li Y. Wang P. (2015). Effects of different *Lactobacillus reuteri* on inflammatory and fat storage in high-fat diet-induced obesity mice model. *J. Funct. Foods* 14 424–434. 10.1016/j.jff.2015.02.013

[B53] Rabiei N. Badi S. A. Marvasti F. E. Sattari T. N. Vaziri F. Siadat S. D. (2019). Induction effects of *Faecalibacterium prausnitzii* and its extracellular vesicles on toll-like receptor signaling pathway gene expression and cytokine level in human intestinal epithelial cells. *Cytokine* 121:154718. 10.1016/j.cyto.2019.05.005 31153056

[B54] Rastelli M. Knauf C. Cani P. D. (2018). Gut microbes and health: a focus on the mechanisms linking microbes, obesity, and related disorders. *Obesity* 26 792–800. 10.1002/oby.22175 29687645 PMC5947576

[B55] Rather S. A. Pothuraju R. Sharma R. K. De S. Mir N. A. Jangra S. (2014). Anti-obesity effect of feeding probiotic dahi containing *Lactobacillus casei* NCDC 19 in high fat diet-induced obese mice. *Int. J. Dairy Technol.* 67 504–509. 10.1111/1471-0307.12154

[B56] Reunanen J. Kainulainen V. Huuskonen L. Ottman N. Belzer C. Huhtinen H. (2015). *Akkermansia muciniphila* adheres to enterocytes and strengthens the integrity of the epithelial cell layer. *Appl. Environ. Microbiol.* 81 3655–3662. 10.1128/AEM.04050-14 25795669 PMC4421065

[B57] Samad F. Yamamoto K. Pandey M. Loskutoff D. J. (1997). Elevated expression of transforming growth factor-β in adipose tissue from obese mice. *Mol. Med.* 3 37–48. 10.1007/BF03401666 9132278 PMC2230108

[B58] Schneeberger M. Everard A. Gómez-Valadés A. G. Matamoros S. Ramírez S. Delzenne N. M. (2015). *Akkermansia muciniphila* inversely correlates with the onset of inflammation, altered adipose tissue metabolism and metabolic disorders during obesity in mice. *Sci. Rep.* 5:16643. 10.1038/srep16643 26563823 PMC4643218

[B59] Shen Y. Torchia M. L. G. Lawson G. W. Karp C. L. Ashwell J. D. Mazmanian S. K. (2012). Outer membrane vesicles of a human commensal mediate immune regulation and disease protection. *Cell Host Microbe* 12 509–520. 10.1016/j.chom.2012.08.004 22999859 PMC3895402

[B60] Singh G. M. Danaei G. Farzadfar F. Stevens G. A. Woodward M. Wormser D. (2013). The age-specific quantitative effects of metabolic risk factors on cardiovascular diseases and diabetes: a pooled analysis. *PLoS One* 8:e65174. 10.1371/journal.pone.0065174 23935815 PMC3728292

[B61] Sousa-Pinto B. Gonçalves L. Rodrigues A. R. Tomada I. Almeida H. Neves D. (2016). Characterization of TGF-β expression and signaling profile in the adipose tissue of rats fed with high-fat and energy-restricted diets. *J. Nutr. Biochem.* 38 107–115. 10.1016/j.jnutbio.2016.07.017 27736730

[B62] Stienstra R. Duval C. Müller M. Kersten S. (2007). PPARs, obesity, and inflammation. *PPAR Res.* 2007:95974. 10.1155/2007/95974 17389767 PMC1783744

[B63] Tabasi M. Ashrafian F. Khezerloo J. K. Eshghjoo S. Behrouzi A. Javadinia S. A. (2019). Changes in gut microbiota and hormones after bariatric surgery: a bench-to-bedside review. *Obesity Surg.* 29 1663–1674. 10.1007/s11695-019-03779-7 30793228

[B64] Torres S. Fabersani E. Marquez A. Gauffin-Cano P. (2019). Adipose tissue inflammation and metabolic syndrome. The proactive role of probiotics. *Eur. J. Nutr.* 58 27–43. 10.1007/s00394-018-1790-2 30043184

[B65] Tsuchida A. Yamauchi T. Takekawa S. Hada Y. Ito Y. Maki T. (2005). Peroxisome proliferator–activated receptor (PPAR) α activation increases adiponectin receptors and reduces obesity-related inflammation in adipose tissue: comparison of activation of PPARα, PPARγ, and their combination. *Diabetes* 54 3358–3370. 10.2337/diabetes.54.12.3358 16306350

[B66] Turnbaugh P. J. Ridaura V. K. Faith J. J. Rey F. E. Knight R. Gordon J. I. (2009). The effect of diet on the human gut microbiome: a metagenomic analysis in humanized gnotobiotic mice. *Sci. Transl. Med.* 1:6ra14. 10.1126/scitranslmed.3000322 20368178 PMC2894525

[B67] Van Baarlen P. Wells J. M. Kleerebezem M. (2013). Regulation of intestinal homeostasis and immunity with probiotic *Lactobacilli*. *Trends Immunol.* 34 208–215. 10.1016/j.it.2013.01.005 23485516

[B68] Wahli W. Michalik L. (2012). PPARs at the crossroads of lipid signaling and inflammation. *Trends Endocrinol. Metabol.* 23 351–363. 10.1016/j.tem.2012.05.001 22704720

[B69] WHO (2016). *Fact Sheet: Obesity and Overweight.* Available at: https://www.who.int/news-room/fact-sheets/detail/obesity-and-overweight (accessed February 16, 2018).

[B70] Yadav H. Quijano C. Kamaraju A. K. Gavrilova O. Malek R. Chen W. (2011). Protection from obesity and diabetes by blockade of TGF-β/Smad3 signaling. *Cell Metab.* 14 67–79. 10.1016/j.cmet.2011.04.013 21723505 PMC3169298

[B71] Zandbergen F. Van Dijk S. Müller M. Kersten S. (2006). Fasting-induced adipose factor/angiopoietin–like protein 4: a potential target for dyslipidemia? *Future Lipidol.* 1 227–236. 10.2217/17460875.1.2.227

[B72] Zhang H. Sparks J. B. Karyala S. V. Settlage R. Luo X. M. (2015). Host adaptive immunity alters gut microbiota. *ISME J.* 9 770–781. 10.1038/ismej.2014.165 25216087 PMC4331585

[B73] Zhao S. Liu W. Wang J. Shi J. Sun Y. Wang W. (2017). *Akkermansia muciniphila* improves metabolic profiles by reducing inflammation in chow diet-fed mice. *J. Mol. Endocrinol.* 58 1–14. 10.1530/JME-16-0054 27821438

